# Critical role for monocytes in mediating HIV-specific antibody-dependent cellular cytotoxicity

**DOI:** 10.1186/1742-4690-9-S2-P51

**Published:** 2012-09-13

**Authors:** M Kramski, GF Lichtfuss, A Schorcht, AP Johnston, R De Rose, R Center, A Jawarowski, S Kent

**Affiliations:** 1 University of Melbourne, Melbourne, Australia; 2 Burnet Institute, Melbourne, Australia

## Background

Antibodies (Abs) that mediate antibody-dependent cellular cytotoxicity (ADCC) activity against HIV-1 are of major interest. Considerable evidence supports a role for ADCC activity in the control of HIV-1 infection and in the context of vaccination. One method widely used to assess the role of ADCC is the rapid and fluorometric antibody-dependent cellular cytotoxicity (RFADCC) assay. In the RFADCC assay specific killing of target cells by PBMC is assessed by loss of intracellular CFSE but retention of membrane dye PKH26 (CFSE-PKH26+), which is assumed to be derived from CFSE+PKH26+ target cells killed by NK cells. We have revisited this assay to assess the role of effector cells in mediating ADCC.

## Methods

Multi-color flow cytometry was used to analyse gp140-pulsed, CFSE and PKH26 double labeled CEM.NKr-CCR5 cells incubated with HIV+ plasma or purified IgG samples (n=57) and co-cultured with PBMC, purified NK cells, or monocytes prepared from healthy donor blood. Effector/target cell interaction was visualized using image stream flow cytometry and live cell imaging.

## Results

Backgating analysis and phenotyping of CFSE-PKH26+ cells identified CD3-CD14+ monocytes as the major effector cell type. This was confirmed for all 57 HIV+ plasma samples tested. Emergence of the CFSE-PKH26+ cell population was observed following co-culture with purified monocytes but not purified NK cells. No significant IFNγ production or CD107a degranulation was detected in NK cells in this assay. Image flow cytometry and microscopy confirmed a monocyte-specific interaction with target cells. Monocytes acquire PKH26+ cell membrane presumably derived from killed target cells without typical morphological changes associated with phagocytosis, suggesting monocyte-mediated ADCC.

## Conclusion

Our studies advance the understanding of the cellular events underlying HIV-specific ADCC. The RFADCC assay primarily reflects Ab-mediated monocyte function and has to be treated with caution in regard to NK cell-mediated ADCC. Further studies on the biological importance of HIV-specific monocyte-mediated ADCC are warranted.

